# The Performance Effect of Scheduled Carbohydrate and Caffeine Intake during Simulated Team Sport Match-Play

**DOI:** 10.3390/nu12071926

**Published:** 2020-06-29

**Authors:** John Keane, Aidan Shovlin, Simon Devenney, Shane Malone, Damien Young, Giuseppe Coratella, Kieran Collins, Marcus Shortall

**Affiliations:** 1 Gaelic Sports Research Centre, Department of Science, Tallaght Campus, Technological University Dublin, 24, D24 FKT9 Dublin, Ireland; shovlin94@hotmail.com (A.S.); simon.devenney@tudublin.ie (S.D.); shane.malone@mymail.ittdublin.ie (S.M.); kieran.collins@tudublin.ie (K.C.); shotall@IRFU.ie (M.S.); 2 Limerick Institute of Technology, Thurles Campus, V94 EC5T Limerick, Ireland; damien.young@hotmail.com; 3 Department of Biomedical Sciences for Health, Università degli Studi di Milano, 20133 Milan, Italy; giuseppe.coratella@unimi.it

**Keywords:** team sports, repeated sprint-ability, ergogenic aids, internal load, aerobic performance

## Abstract

The aim of the current investigation was to identify the effects of scheduled carbohydrate (CHO) and caffeine (CAF) supplementation on simulated team sport match-play performance. Ten male hurling players completed three hurling match-play simulation protocols (HSP) performed 7 days apart in a double-blind, randomized design. Supplementation included CHO, CHO + CAF, and placebo (PLA). In a randomized order, participants ingested either a 6% CHO solution, a PLA solution of similar taste, or a combined intake of 6% CHO solution + 200 mg CAF capsule. At specific time points (Pre-0 min; half time (HT)-30 min; full time (FT)-60 min), participants completed a repeated sprint protocol (RAST; 12 × 20 m). Physiological [% maximal oxygen uptake (%VO_2max_), % mean oxygen uptake (%VO_2mean_), % maximal heart rate (%HR_max_), % mean heart rate (%HR_mean_), respiratory exchange ratio (RER), and blood lactate (BLa)] and performance [(best sprint time (RSA_best_), mean sprint time (RSA_mean_), and rate of perceived exertion (RPE)] variables were monitored throughout each simulation. Non-significant differences were observed between supplement trials (CHO, CHO + CAF, and PLA) for BLa (η^2^ = 0.001, *small*), %VO_2max_ (η^2^ = 0.001, *small*), %VO_2mean_ (η^2^ = 0.004, *small*), %HR_max_ (η^2^ = 0.007, *small*), %HR_mean_ (η^2^ = 0.018, *small*), RER (η^2^ = 0.007, *small*), RPE (η^2^ = 0.007, *small*), and RSA_best_ (η^2^ = 0.050, *small*). RSA_mean_ performance significantly improved in CHO + CAF trials compared to PLA, with sprint times significantly improved from Pre to FT also (η^2^ = 0.135, *medium*). A significant difference was observed in BLa between time points (Pre, HT, and FT) (η^2^ = 0.884, *large*) in % HRmax (η^2^ = 0.202, *medium*), %HR_mean_ (η^2^ = 0.477, *large*), and RER (η^2^ = 0.554, *large*) across halves and in RPE across time points (η^2^ = 0.670, *large*). Our data provide novel data regarding the effects of CHO and CAF supplementation on team sport performance, with co-ingestion of CHO + CAF reducing the decrement in repeated sprint performance compared to PLA.

## 1. Introduction

Hurling is a stick and ball intermittent field sport where the ebb and flow of competitive play are superimposed on bouts of high-speed running followed by active recovery [1]. The match-play demands of hurling have been quantified [2,3] with mean total distances (TD) of 7617 ± 1219 m reported for players. Furthermore, players can be expected to cover 15% of match-play distance at high speed (HSD; m; >17 km·h^−1^; 1134 ± 358 m) and 4% sprinting distance (SD; m; >22 km·h^−1^; 319 ± 129 m). It was previously identified that position directly influences the work-rate profiles observed during hurling match-play with midfielders (MF), completing significantly more TD and HSD than other positional lines of play [full backs (FB), half backs (HB), half forwards (HF), and full forwards (FF)] [2]. Interestingly, high-speed work rate was shown to decrease throughout match-play, most notably at the latter stages of each half [2,4], with a distinctive positional trend also present. The largest decrement in HSD was observed in half forwards (27%) followed by half backs (24%), full forwards (23%), midfielders (22%), and full backs (13%). Previous literature has also outlined the anthropometric and performance profiles of elite hurlers with mean anthropometric profiles reported for a sum of five skinfolds (40.1 ± 5.6 mm) and adipose tissue percentage (12.7 ± 8.0%) [5]. Additionally, mean performance profiles were reported for counter movement jump (CMJ) (47.2 ± 5.1 cm), CMJ peak power (4464 ± 490 Watts), sprint performance [5 m (1.00 ± 0.64 s), 10 m (1.86 ± 0.04 s), and 20 m (3.03 ± 0.06 s)], and estimated maximal oxygen uptake (56.3 ± 2.9 mL·kg^−1^) [5].

Previous literature has shown the positive effects associated with the ingestion of carbohydrate (CHO) and caffeine (CAF) within intermittent team sports [6]. CHO ingestion has been observed to reduce the rate of glycogen depletion and maintain blood glucose concentration [7], while CAF has been observed to delay the onset of fatigue [6]. However, it appears that the combined ingestion of CHO and CAF increased the work completed during a 120 min cycling simulation by 23% when compared to placebo and CHO only [8]. More recently, co-ingestion of a CHO + CAF drink (40 g h^−1^ + 3 mg·kg^−1^) was shown to improve the HSD completed and mean sprint speeds of elite rugby league players compared to CHO [9]. The timing and quantity of administered CHO and CAF during research studies has been shown to vary, with recent research suggesting peak caffeine concentration is attained within 1–3 h of ingestion [6]. Administered amounts of 5–6 mg·kg^−1^ CAF have been advised to produce ergogenic effects during exercise [10]. CHO, however, is often administered prior to and at specific intervals during exercise. Administered amounts typically reflect 5 mL·kg^−1^ of 6–7% solution provided prior to exercise, with an additional 2 mL·kg^−1^ provided at regular intervals during exercise [11].

While the performance enhancing effects of caffeine have been investigated for over 100 years [12], the specific mechanisms of action are still debated to date. Caffeine is rapidly absorbed through the gastrointestinal tract and metabolized in the liver by cytochrome P450 1A2 (CYP1A2) [13]. Previously, the primary mechanism of action was related to the antagonism of adenosine receptors in the brain [14]. Additional mechanisms outlined include the inhibition of phosphodiesterase’s, where the accumulation of cyclic adenosine monophosphate (AMP) and increased levels of catecholamines illicit cognitive responses such as increased alertness and attention capacity [15]. Recent research has also identified potential individual responses to caffeine dependent on genetic variation [16,17]. These modern investigations suggest that individuals may be categorized into responders and non-responders to the ergogenic effects of caffeine. Specifically, two single nucleotide polymorphisms (SNPs), CYP1A2 and ADORA2A, have received considerable attention in recent literature [18]. It has been suggested that moderate doses of caffeine (4 mg·kg^−1^) can be ergogenic in AA genotypes, ergolytic in CC genotypes, while having a minimal effect for AC genotypes [18]. Potential reasoning includes the AA genotype being associated with increased production of the enzyme P450 1A2, resulting in quicker metabolization of caffeine [19]. Further research is required to validate these mechanisms, yet it could lead to greater understanding in relation to the specific utilization of caffeine supplementation within participant groups to optimize the performance of those who are more likely to have a positive response to the ingestion of caffeine. 

One of the challenges faced by a practitioner when aiming to apply previous research findings is that several studies investigating the effects of CHO do so in a fasted state [20]. The aforementioned methodology compromises the ecological validity of interventions due to the nutritional intake and pre-competition preparatory practices completed by athletes, which is focused towards optimal fueling for performance [21], with current carbohydrate intake recommendations the day before a game of 7 g kg^−1^ and further supplementation of 1–4 g kg^−1^ recommended 1–4 h pre competition [22]. Given the above, the aim of the current investigation was to identify the effects of scheduled carbohydrate (CHO) and caffeine (CAF) supplementation on simulated team sport match-play performance. The current study is the first to apply a nutritional intervention within a hurling cohort with the aim to determine the effect of CHO and CAF ingestion prior to and during a hurling match-play simulation protocol. Given that nutritional intervention investigations completed in other intermittent field sports have shown performance benefits from co-ingestion of CHO + CAF, it would appear that there is the potential for such nutritional interventions to have a positive effect on hurling match-play performance. It was hypothesized that the ingestion of CHO and CAF would present performance benefits when compared to identical volumes of placebo content during simulated match-play.

## 2. Materials and Methods 

### 2.1. Participants

Ten (*n* = 10) male Caucasian sub-elite hurling players (mean ± SD age; 22 ± 2 years, height; 1.72 ± 0.04 m, body mass; 70.7 ± 8.2 kg, maximal oxygen uptake (VO_2max_); 56.8 ± 4.3 mL·kg^−1^·min^−1^) were recruited for the current study. Written consent was provided by all participants to the experimental procedures following a briefing on all possible benefits and risks of the study. Participants were instructed to refrain from alcohol, caffeinated food, caffeinated beverages, and vigorous exercise for 48 h prior to laboratory testing and the match-play simulation. Participants were instructed to adhere to a CHO loading protocol for 48 h prior to testing, as the study was designed to be completed in a fed state [23]. The CHO loading protocol provided outlined meal options for breakfast, lunch, dinner, and snacks. Participants were instructed to consume 5–6 g·kg^−1^ of CHO and 1.7 g·kg^−1^ of protein as per research guidelines for exercise less than 90 min in duration [22]. Ethical clearance was approved by the local institutions research ethics committee and conducted according to the Declaration of Helsinki (1975) for studies involving human subjects.

### 2.2. Preliminary Measurements

Participants’ anthropometric measurements were recorded using a stadiometer and weighing scales (Detecto, Webb City, MS, USA), respectively. VO_2max_ testing was completed prior to the match-play simulations. The VO_2max_ assessment comprised of five 4 min stages at increasing speeds (8, 10, 12, 14, and 16 km·h^−1^), followed by an incremental ramp to exhaustion of 1 km·min^−1^ until the participant reached exhaustion [1]. To best reflect outdoor running, the gradient of the treadmill was set at 1% for the entire test [24]. One minute rest intervals separated each 4 min stage to allow for a fingertip capillary blood lactate sample to be obtained (Lactate Plus, Nova Biomedical, Waltham, Massachusetts, USA). VO_2max_ and respiratory exchange ratio (RER) were recorded using a breath by breath gas analyzer (Cosmed K4b2, Cosmed, Rome, Italy). VO_2max_ was recorded as the maximum mean VO_2_ that was attained during a 1 min period of the testing procedure with two of the following parameters achieved; (1) RER of >1.10; (2) a plateau in VO_2_ despite the increase in treadmill speed; (3) participant achieved age predicted maximum heart rate (MHR) [25]. Heart rate (HR) was continuously monitored throughout the test using Heart Rate belt monitors (Polar T31, Polar Electro, Kempele, Finland). MHR recorded along with VO_2max_ calculated during the test provided reference values to determine % maximal heart rate (%HR_max_) and % maximal oxygen uptake (%VO_2max_) attained by each participant during the Hurling Simulation Protocols (HSPs).

### 2.3. Experimental Protocol

**Figure 1:**
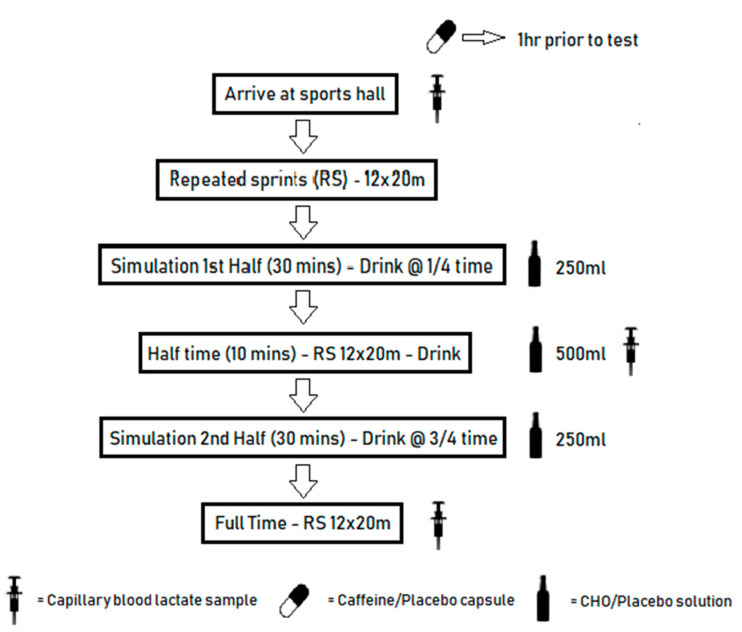
Schematic outline of hurling simulation protocols (HSP) supplementation design [carbohydrate (CHO)].

During weeks 2–5, participants completed three HSP with specific supplementation [CHO, CHO + CAF, and placebo (PLA)], each separated by a 5-day recovery period (Figure 1). Experimental trials were completed in randomized, double blinded order and all trials were completed at a similar time of day (10:00–15:00) to avoid circadian variations in performance [26]. All participants completed one simulation protocol under each of the supplement conditions (CHO, CHO + CAF, and PLA). Participants did not receive the same supplement more than once. The HSP [27] was selected due to its accurate physiological replication of hurling match-play. Reliability and external validity of the HSP had been previously confirmed [27]. Within this reliability study, participants completed an average relative TD of 110 ± 2 m·min^−1^ and relative HSD (m; >17 km·h^−1^) of 19 ± 2 m min^−1^ [27]. Recent analysis of competitive match-play at an elite level reported that players completed an average relative TD of 108 ± 17 m·min^−1^ and relative HSD (m; >17 km·h^−1^) of 16 ± 5 m·min^−1^ 1 [2]. Each participant completed a 10 min standardized warm-up prior to commencement of HSP. The HSP consisted of two 30 min halves, comprised of multiple movements (walk, back pedal, shuffle, cruise, and sprint) separated by a 10 min half time interval.

A repeated sprint protocol (12 × 20 m maximal shuttles) [28] was completed by each participant prior to, at half time (HT), and at full time (FT) of the HSP to assess the temporal profile of repeated sprint ability (RSA). Mean (RSA_mean)_ and best (RSA_best_) shuttle sprint times were recorded during repeated sprints protocol using a timing gate system (Witty Timing System, Microgate, Bolzano, Italy). VO_2max_ and RER were recorded using a breath by breath gas analyzer (Cosmed K4b2, Cosmed, Roma, Italy). HR was continuously monitored throughout all stages of the trials using the Polar Electro HR belt monitor (Polar T31, Polar Electro, Kempele, Finland). For the purposes of this study, percentage average and maximum values for VO_2_ and HR were reported across halves to determine variation between halves.

Blood lactate measurements were obtained at three stages during each protocol; resting lactate concentration was measured prior to commencement of repeated sprints and HSP, at half time, and at full-time. Blood lactate (Bla) measurements were obtained at three stages during each protocol; resting lactate concentration was measured prior to commencement of HSP, at HT, and at FT. Lactates were measured using the fingertip capillary method (Lactate Plus, Nova Biomedical, Waltham, Massachusetts, USA). Rate of perceived exertion (RPE) was recorded using the 6–20 Borg Scale [29] at 10 six-minute interval stages (S1–S10) during the HSPs.

### 2.4. Supplement Administration

CHO supplementation was administered at specific intervals (15, 30, 45 min) throughout both the CHO and CHO + CAF trials. The CHO supplement was administered at standard time intervals; 250 mL at 15 min, 500 mL at 30 min, and 250 mL at 45 min of the HSP. CHO supplementation was a 6% maltodextrin solution [30]. CAF supplementation was administered for the CHO + CAF trial in a capsule containing 200 mg 1 h prior to commencement of testing [31], while a non-caffeinated capsule was administered prior to the commencement of the CHO and PLA trials. During the PLA trial, a non-CHO solution of a similar color and taste was administered at precisely the same times as other trials. In contrast to previous literature, we decided not to include a CAF-only trial condition. In line with common preparatory routines of players prior to team sport match-play, which are based considerably around CHO intake [32], we believed the combination of CHO + CAF enhanced the ecological validity of this study in relation to competitive match-play.

### 2.5. Statistical Analysis

All statistical analyses was performed using the Statistical Package for Social Sciences software (SPSS Version 23.0, Chicago, IL). Results are presented as descriptive statistics (means ± standard deviation (SD), 95% confidence intervals (95% CI)) unless stated. Partial eta squared (*η*^2^) was reported and defined as small 0.02–0.12, medium 0.13–0.25, and large >0.26 [33], with statistical significance set at *p* < 0.05. A preliminary analysis was carried out to ensure normal distribution within the data (Shapiro Wilks test). Further analysis was completed to detect major outliers (greater than three standard deviations), which were subsequently removed. Homogeneity of variance within the data were analysed using Levene’s test with (*p* > 0.05). Data were analysed using a two-way (time × trial) analysis of variance (ANOVA), with repeated measures for correlated data. The Greenhouse-Geisser correction was applied when the assumption of sphericity was violated. A Bonferroni post hoc correction was performed to identify statistical significance within the main effects for physiological and performance responses due to time and/or trial interaction effects.

## 3. Results

### 3.1. Physiological Variables 

Differences in physiological variables across time and trial are shown as means ± SD, 95% CI, with main condition effects and effect sizes for time and trial conditions also reported (Table 1 and Table 2). Non-significant differences were observed between supplement trials (CHO, CHO + CAF, and PLA) across BLa (*p* = 0.981; η^2^ = 0.001, *small*), %VO_2max_ (*p* = 0.985; η^2^ = 0.001, *small*), %VO_2mean_ (*p* = 0.949; η^2^ = 0.004, *small*), %HR_max_ (*p* = 0.912; η^2^ = 0.007, *small*), %HR_mean_ (*p* = 0.787; η^2^ = 0.018, *small*), and RER (*p* = 0.933; η^2^ = 0.005, *small*), respectively. Conversely, differences were reported across variables between time conditions. Specifically, significant differences were reported for BLa between time conditions (Pre, HT, and FT) (*p* = 0.001; η^2^ = 0.844, *large*), with a significant increase observed relative to FT at pre (*p* = 0.001) and HT (*p* = 0.001), while also from pre to HT (*p* = 0.001) (Table 2). A significant decrease in %HR_max_ (*p* = 0.014; η^2^ = 0.202, *large*), %HR_mean_ (*p* = 0.001; η^2^ = 0.408, *large*), and RER (*p* = 0.001; η^2^ = 0.554, *large*) was reported across halves (Table 1).

### 3.2. Performance Variables 

**Figure 2:**
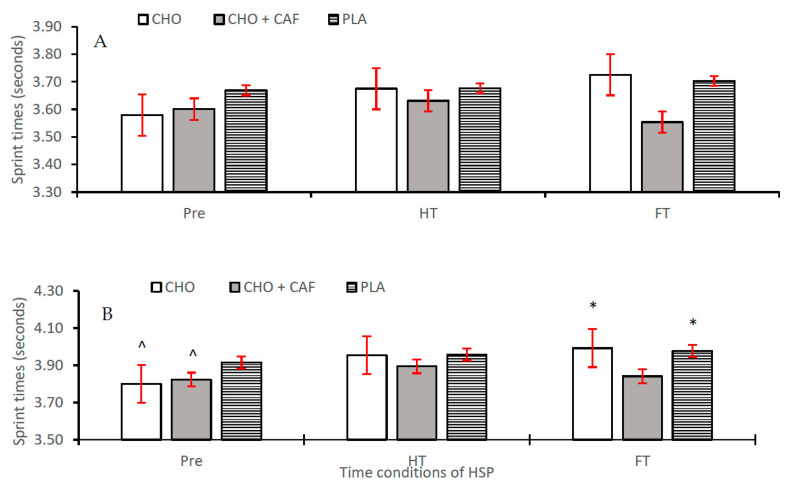
RSA_best_ (**A**) and RSA_mean_ (**B**) recorded at each time condition (Pre, half time (HT), and full time (FT) across all supplement trials [carbohydrate (CHO), placebo (PLA), and CHO + caffeine (CAF)]) throughout hurling simulation protocols (HSP). Mean ± standard deviation (SD). * = significantly different from CHO + CAF. ^ = significantly different from PLA.

**Figure 3:**
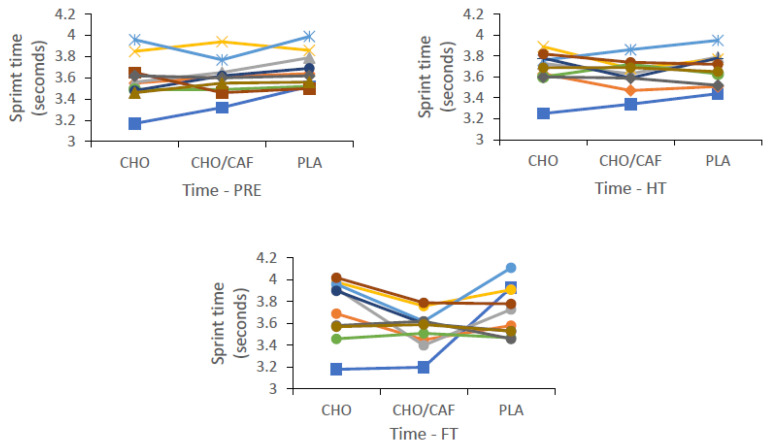
Individual best sprint (RSA_best_) differences at each time condition [Pre, half time (HT), and full time (FT)] across all supplement trials [carbohydrate (CHO), placebo (PLA), and CHO + caffeine (CAF)] throughout hurling simulation protocols (HSP). [Means ± standard deviation (SD)].

**Figure 4:**
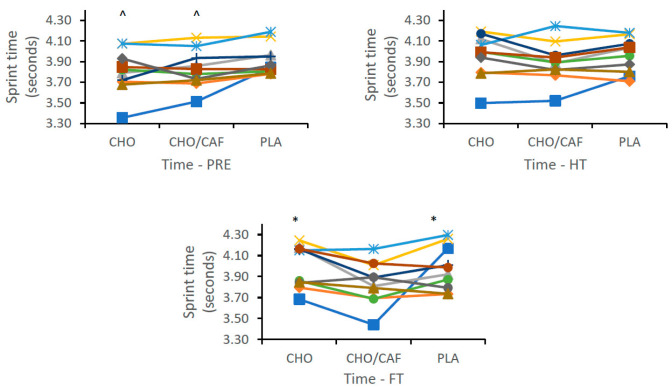
Individual mean sprint (RSA_mean_) differences at each time condition [Pre, half time (HT), and full time (FT)] across all supplement trials [carbohydrate (CHO), placebo (PLA), and CHO + caffeine (CAF)] throughout hurling simulation protocols (HSP). [Means ± standard deviation (SD)]. * = significantly different from CHO + CAF. ^ = significantly different from PLA.

**Figure 5:**
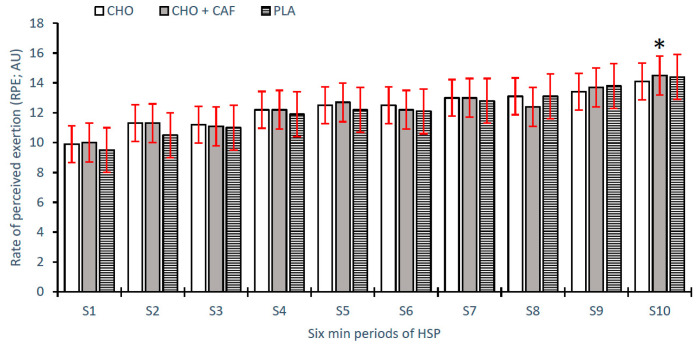
Rating of perceived exertion (RPE) at each six-minute period (S1-S10) of hurling simulation protocol (HSP) across all supplement trials [carbohydrate (CHO), placebo (PLA), and CHO + caffeine (CAF)]. (Mean ± standard deviation (SD). * = significantly different from S1–S8.

Differences in performance variables across time and trial are shown as means ± SD, 95% CI, with main condition effects and effect sizes for time and trial conditions also reported (Table 2). There was a non-significant difference reported between supplement trials (CHO, CHO + CAF, and PLA) for RPE (*p* = 0.914; η^2^ = 0.007, *small*) and RSA_best_ (*p* = 0.502; η^2^ = 0.050, *small*), respectively. Concurrently, a significant difference was reported regarding RSAmean between supplement trials (*p* = 0.002; η^2^ = 0.028, *small*), with a significant difference reported between CHO + CAF and PLA trials. Variations in sprint performance (RSA_mean_) between trials were also evident across time. Significant differences were seen at pre (*p* = 0.001; η^2^ = 0.044, *small*), with CHO and CHO + CAF trials significantly different to PLA (*p* = 0.001; *p* = 0.008), and at FT (*p* = 0.001; η^2^ = 0.066, *medium*), with sprint times in the CHO + CAF trial significantly reduced compared to the CHO and PLA trials, respectively (Figure 2). Individual differences across participants for best and mean sprint times are shown in Figure 3 and Figure 4, respectively. Finally, a main condition effect (*p* = 0.001; η^2^ = 0.670, *large*) showed a significant increase in RPE from the first 6 min stage (S1) to the final 6 min interval recorded (S10). Differences between stages (S1–S10) can be observed in Figure 5.

## 4. Discussion

The aim of the current investigation was to assess the effects of scheduled CHO and CAF ingestion both prior to and during simulated hurling match-play. We provide novel data on the effects of such supplementation during match-play specifically, showing that supplementation (CHO, CHO + CAF, and PLA) did not significantly impact %VO_2max_, %VO_2mean_, RER, %HR_max_, and %HR_mean_. Interestingly, %HR_max_, %HR_mean_, and RER decreased across the halves of play, independent of the supplement provided. Furthermore, a significant improvement in RSA_mean_ was observed at pre-test following CHO and co-ingestion of CHO and CAF when compared to the PLA. Additionally, these effects were also observed at full time following co-ingestion of CHO and CAF, but not CHO and PLA, with RSA_best_ not significantly altered. Finally, BLa and RPE significantly increased across time conditions, with no impact observed for any of the supplements. The current data provides novel information for practitioners to improve match-play performance, suggesting that the combined utilization of CHO + CAF may improve repeated sprint ability within hurling cohorts.

Our data indicated that VO_2_, RER, and HR responses were not significantly affected in response to nutritional intervention. Specifically, during the first half, the %VO_2max_ attained with CHO (76 ± 7%), CHO + CAF (75 ± 7%), and PLA (76 ± 5%) were not significantly different. During the second half, an increase of 1% was shown across the CHO trial (76 ± 8%) in comparison to the CHO + CAF (75 ± 7%) and PLA (75 ± 6%) trials, respectively. Marginal differences in VO_2_ values were also reported in previous studies [34]. Furthermore, a significant decrease in RER was reported during the second half (0.79 ± 0.12) compared to the first (0.85 ± 0.11), indicating a possible depletion of glycogen during match-play resulting in a shift in the level of CHO utilization during the second half of match-play running performance [35]. RER was not significantly affected depending on supplement, which is in line with previous literature [34,36]. These observations suggest that administering CHO and CAF may not be effective in increasing the rate of glycogen sparing throughout simulated match-play activities. Similarly, authors [36] showed that while depletion of muscle glycogen occurred throughout match-play, differences were not evident between supplement conditions (3 g kg^−1^–6 g kg^−1^ CHO, respectively). Therefore, it would seem that the improvement in RSA performance during the current study is independent of any effect of CAF on substrate utilization and is more likely through a direct effect on the CNS [37] resulting in improved RSA performance during the CHO + CAF trial.

A reduction in %HR_max_ and %HR_mean_ was observed across halves independent of supplementation. Previously, Scott and colleagues [34] reported minimal change in HR responses between pre- and post-exercise independent of supplementation, with a limited effect of CHO + CAF on HR responses in comparison to CHO during a 2000 m rowing simulation. However, our results are in contrast to Gant et al. [38], who identified a significant difference in HR responses between trials where CHO + CAF increased HR in the later stages of simulated soccer match-play compared to CHO. A possible rationale for the decrease in HR across halves in the current study is that as exercise increases, the impact of fatigue will result in a suppression of HR responses over time [39,40]. To further support the presence of fatigue, a significant increase in RPE was reported over time, therefore implying that increased exertion is complemented by the suppression of HR during the latter stages of match-play. Given the above information, it appears apparent that despite specific nutritional interventions, several mechanisms resulted in an element of physiological fatigue. These mechanisms include the depletion of glycogen stores, since development of fatigue during prolonged intermittent exercise has been associated with a lack of muscle glycogen [41], an increase in RPE, and a decrease in HR response over time, indicating a suppression of internal response to a given work-rate.

RSA has previously been reported as an important physical quality with regard to hurling match-play performance [4]. RSA has been identified as a determining factor in goal scoring situations during soccer match-play, with 45% of all analysed goals scored preceded by a bout of RSA [42]. We observed a significant difference in RSA_mean_ between supplement trials (*p* = 0.002; η^2^ = 0.033, *small*), with a significant difference reported between CHO + CAF trials and PLA trials (*p* = 0.002) specifically. RSA_best_ times were not improved following interventions. Variations in sprint performance between trials were also evident across time conditions (Table 2). Previous research indicates a variation in the potential impact of CAF on RSA performance depending on study design and trial conditions. However, there is considerable research to support a beneficial hypothesis. Gant et al. [38] indicated that there was a significant improvement in 15 m sprint performance during the CHO + CAF trial compared to CHO only. Similar to our results, performance improvements were apparent during the later stages of the RSA testing protocol. While certain simulation studies have also determined a lessened effect on sprint performance following nutritional supplementation [43], it would appear that RSA performance during hurling match-play can be improved following co-ingestion of CHO + CAF supplementation.

A temporal profile was observed for RPE across match-play, with significant increases at S10 compared to S1–S8. These results indicate that as duration of exercise increased, there was a linear increase in the perception of fatigue. Likewise, BLa was significantly increased at full time compared to both pre and half time, independent of trial supplements. Neither RPE nor BLa were significantly altered based on supplement treatments. Previous literature has reported similar increases in BLa throughout testing protocols, but not between trials [38,44]. Contrary to this, Scott et al. [34] also presented minimal difference between trials, while differences pre- and post-testing showed no significant differences. RPE was shown to significantly increase as testing progressed, however not between supplement treatments, indicating that despite nutritional supplementation, no effect was observed on player’s perception of fatigue across match-play [10,38,43,44]. However, the improvements in RSA_mean_ performance at FT during the CHO + CAF treatment suggest that repeated running performance decrements may be limited as a result of nutritional supplementation during match-play. 

The present investigation is not without limitations. Firstly, although all participants were advised to adhere to a CHO loading protocol for 48 h prior to each HSP, adherence was verbally assessed without the inclusion of food intake logs. While this may be considered a limitation due to the potential for participant bias within responses, the participants recruited were from a sub-elite cohort of players who were unfamiliar with the process of completing food intake logs. Given the amateur nature of players, they may have failed to complete reliable food intake logs, therefore verbal assessment was potentially a more ecologically valid method of assessment. It is also impractical for coaches to implement food intake logs in a sub-elite environment and as such, this method of adherence assessment provided a practical reflection of players pre-competition adherence to fuelling. Furthermore, in contrast to a number of studies, an absolute caffeine dosage was administered (200 mg) instead of dosages relative to individual participant body mass, possibly contributing to variations in results [31]. This aspect of the study could also be viewed as an ecological strength as dosages in readily available caffeinated supplements are rarely prescribed based on athlete body mass. Additionally, due to the nature of team sport environments, it is more practically relevant to consider standardized dosages as the preparation of individual caffeine dosages based on body mass is not reflective of the time available to practitioners. While the concept of caffeine tolerance can also attenuate an individual’s responses to supplementation, previous literature has reported continued performance benefits following prolonged caffeine supplementation (15–18 days) [45]. It was reported that the magnitude of the effect suggested a progressive tolerance over time. Further research is required to definitively ascertain the impact of caffeine tolerance in relation to the performance benefits associated with supplementation. Finally, it should be noted that positional differences are not accounted for within the HSP, therefore the distance outlined in the HSP represents an average distance covered between the three central positional lines of half backs, midfielders, and half forwards. This limits the individual demands placed on participants as full backs and full forwards have been reported to cover a reduced sum of total distance and high intensity distance compared to the other positional lines [2]. 

Potential practical applications arising from the current study include improved repeated sprint ability following CHO + CAF supplementation. It is well documented that the transitional lines of the field, half backs, midfield, and half forwards complete greater amounts of total and high intensity work compared to the inside lines [2] and as such, are expected to produce an increased number of repeated sprint efforts throughout match-play. Our findings suggest that players within these positions may look to CHO + CAF supplementation to maintain their ability to complete repeated sprint efforts as duration increases. Repeated sprint performance is also a key performance outcome in relation to other high intensity intermittent field sports such as Gaelic football and soccer [42,46]. Considering that CHO + CAF supplementation can potentially improve repeated sprint ability in hurling, these finding may translate appropriately for practitioners involved within these sports. Previous literature has also outlined the concept of individuals being identified as responders vs non-responders in relation to the erogenicity of caffeine [47]. Practitioners may trial the use of caffeine within intermittent team sport environments, however where performance benefits are minimal or where individuals display ergolytic responses, then it may be of benefit to cease supplementation. Caffeine is commonly administered in two forms within team sports—gum and caffeinated drink supplementation. As such, the idea of research-supported combined CHO + CAF supplementation in a hurling cohort is an important implication for practitioners. CAF was administered 1 h prior to competition, which is easily replicable in a team sport setting as players will usually arrive 1 h prior to games. CHO supplementation can be administered in gels or liquid solution in the form of water bottles throughout the game. This study has shown that administration at the end of each quarter of the game (15, 30, 45 min) provides potential benefits. Finally, consistent with the pre-match preparations for athletes, this study was completed in a fed state and as such, the findings have practical implications for hurling match-play.

## 5. Conclusions

The present study is the first to investigate the effects of nutritional interventions in hurling. Consistent with the pre-match preparations for athletes, this study was completed in a fed state and as such, the findings have practical implications for hurling match-play. Combined ingestion of CHO + CAF had a significant effect on sprint performance specifically at full time, with an apparent decrease in performance decrements compared to CHO alone and PLA as the influence of fatigue appeared to be reduced at the end of the simulation protocol. This study provides normative data whereby coaches, practitioners, or players themselves may choose to implement supplementation prior to and/or during competitive match-play to improve sprint performance. 

## Figures and Tables

**Table 1:** Physiological responses across halves (first & second) of match-play for each supplement trial [carbohydrate (CHO), placebo (PLA), CHO + caffeine (CAF)]. Mean ± standard deviation (SD) and 95% confidence intervals) (95% CI). * = significant difference between time conditions.

Variable	Trial	1st Half	2nd Half	Trial (*p*), η^2^	Time Effect (*p*), η^2^
%VO2_max_	CHO	76 ± 7 (71–80)	76 ± 8 (71–81)		
	CHO + CAF	75 ± 7 (71–80)	75 ± 7 (70–80)	0.985, 0.001, small	0.621, 0.009, small
	PLA	76 ± 7 (72–81)	75 ± 6 (70–79)		
%VO2_mean_	CHO	73 ± 8 (69–78)	73 ± 7 (69–78)		
	CHO + CAF	73 ± 7 (68–77)	73 ± 6 (68–77)	0.949, 0.004, small	0.856, 0.001, small
	PLA	72 ± 6 (68–77)	72 ± 7 (68–77)		
%HR_max_	CHO	89 ± 6 (85–94)	87 ± 5 (83–90)		
	CHO + CAF	89 ± 6 (85–93)	86 ± 7 (82–90)	0.912, 0.007, small	0.014 *, 0.202, medium
	PLA	90 ± 6 (85–93)	87 ± 5 (84–91)		
%HR_mean_	CHO	79 ±5 (76–82)	78 ± 5 (76–81)		
	CHO + CAF	79 ± 5 (77–82)	77 ± 5 (75–80)	0.787, 0.018, small	0.001 *, 0.408, large
	PLA	79 ± 4 (76–82)	76 ± 4 (74–83)		
RER	CHO	0.86 ± 0.10 (0.79–0.93)	0.79 ± 0.11 (0.72–0.87)		
	CHO + CAF	0.85 ± 0.12 (0.78–0.92)	0.79 ± 0.12 (0.72–0.87)	0.933, 0.005, small	0.001 *, 0.554, large
	PLA	0.85 ± 0.12 (0.77–0.92)	0.77 ± 0.13 (0.69–0.85)		

**Table 2:** Physiological and performance data across time conditions (Pre, half time (HT), and full time (FT) during match-play for each supplement trial [carbohydrate (CHO), placebo (PLA), and CHO + caffeine (CAF)]. Mean ± standard deviation (SD) and 95% confidence intervals) (95% CI). * = significant difference between time conditions.

Variable	Trial	Pre	HT	FT	Trial (*p*), η^2^	Time Effect (*p*), η^2^
BLA (mmoL L^−1^)	CHO	1.4 ± 0.7 (1.0–1.8)	5.5 ± 1.8 (4.0–6.3)	7.8 ± 2.2 (6.5–9.1)		
	CHO + CAF	1.5 ± 0.6 (1.1–1.9)	5.0 ± 1.4 (3.9–6.1)	7.9 ± 1.9 (6.6–9.2)	0.981, 0.001, small	0.001 *, 0.884, large
	PLA	1.5 ± 0.3 (1.1–1.8)	4.7 ± 1.4 (3.6–5.9)	8.0 ± 1.8 (6.7–9.3)		
RSA_mean_(s)	CHO	3.80 ± 0.21 (3.76–3.84)	3.95 ± 0.21 (3.91–4.00)	3.99 ± 0.20 (3.95–4.04)		
	CHO + CAF	3.82 ± 0.18 (3.78–3.87)	3.89 ± 0.19 (3.85–3.94)	3.84 ± 0.21 (3.79–3.89)	0.002 *, 0.033, small	0.001 *, 0.135, medium
	PLA	3.91 ± 0.15 (3.87–3.96)	3.96 ± 0.17 (3.92–4.00)	3.98 ± 0.21 (3.93–4.02)		
RSA_best_ (s)	CHO	3.58 ± 0.22 (3.46–3.70)	3.68 ± 0.18 (3.57–3.78)	3.73 ± 0.28 (3.58–3.87)		
	CHO + CAF	3.60 ± 0.17 (3.48–3.72)	3.63 ± 0.15 (3.53–3.74)	3.55 ± 0.17 (3.41–3.70)	0.502, 0.050, small	0.220, 0.055, small
	PLA	3.67 ± 0.16 (3.55–3.79)	3.68 ± 0.16 (3.57–3.78)	3.70 ± 0.22 (3.55–3.85)		
